# IRE1α Expedites the Progression of Castration-Resistant Prostate Cancers *via* the Positive Feedback Loop of IRE1α/IL-6/AR

**DOI:** 10.3389/fonc.2021.671141

**Published:** 2021-07-06

**Authors:** Fan Yang, Chong Yuan, Dan Wu, Jing Zhang, Xingchun Zhou

**Affiliations:** ^1^ Department of Physiology and Pathophysiology, State Key Laboratory of Cancer Biology, Air Force Medical University, Xi’an, China; ^2^ Department of Urology, Tangdu Hospital, The Air Force Medical University, Xi’an, China; ^3^ Department of Clinical Laboratory, XiJing Hospital, The Air Force Military Medical University, Xi’an, China; ^4^ Department of Microbiology and Immunology, Medical School of Yan’an University, Yan’an, China; ^5^ Experimental Teaching Center of Basic Medicine, The Air Force Military Medical University, Xi’an, China

**Keywords:** castration-resistant prostate cancer, IRE1α, IL-6, androgen receptor, positive-feedback loop

## Abstract

Castration-resistant prostate cancer (CRPC) is the lethal form of prostate cancer (PCa), and the underlying molecular mechanism has not been fully elucidated. Inositol requiring enzyme 1 alpha (IRE1α), a key regulator of unfolded protein response (UPR), is intimately associated with PCa progression. However, whether IRE1α is implicated in CRPC development remains unknown. Here, we showed that IRE1α expression was significantly increased in CRPC tissues and high-grade PCa tissues. Overexpression of IRE1α promoted PCa cell proliferation under the androgen deficiency condition *in vitro* and *in vivo*. Mechanistically, increased IRE1α expression induced IL-6 secretion *via* the IRE1α/XBP-1s signal pathway. IRE1α-induced IL-6 activated androgen receptor (AR), and the activation of AR by IL-6, in turn, promoted IRE1α expression. IRE1α formed a positive feedback loop with IL-6 and AR to promote prostate cancer cell proliferation under the androgen-deficient condition. In clinical PCa samples, high IRE1α expression correlated with elevated IL-6 and increased PSA expression. Our findings demonstrated a novel mechanism of CRPC progression and suggest targeting IRE1α may be a potential target for the prevention and treatment of CRPC.

## Introduction

Prostate cancer (PCa) is the most common malignancy in men (1). As for androgen-dependent cancer, androgen-receptor signaling plays a crucial role in PCa development and progression (2). Androgen deprivation therapy (ADT), achieved through surgical or pharmacological approaches is the standard first-line treatment for advanced prostate cancers. However, after the initial treatment response, a large number of patients will inescapably progress to castrate-resistant prostate cancer (CRPC), which is fatal and has no effective treatment. Extensive studies indicate that CRPC continues to rely on androgen receptor signals despite the availability of only castrate levels of androgens. Reactivation of the androgen receptor (AR) is still regarded as the leading cause of CRPC (3). Mechanisms likely to be involved in the AR reactivation have been studied extensively but have failed to yield meaningful and useful targets (4). Therefore, further elucidating the potential molecular mechanisms underlying ligand-independent AR activation would be an urgent need to develop more effective therapies for CRPC.

Inflammation plays an essential role in the pathogenesis and progression of PCa by producing inflammatory cytokines (5). Interleukin (IL)‐6 is well recognized as a significant regulator of PCa progression of all cytokines. Both IL-6 and its receptor have been shown to be highly expressed in PCa tissues and cell lines (6). IL-6 has also been reported to induce AR activation in a ligand-independent manner. Furthermore, IL‐6‐induced AR activation has been displayed to play a vital role in CRPC progression. IL-6 can facilitate androgen-dependent PCa cell proliferation under androgen deprivation conditions *in vitro* and *in vivo*, with a concomitant increase in androgen‐responsive genes prostate-specific antigen (PSA) expression (7).

Inositol requiring enzyme 1 alpha (IRE1α) is the most important mediator of endoplasmic reticulum (ER) stress-induced the unfolded protein response (UPR), which possess both protein kinase and endoribonuclease activities (8). The function of IRE1α has been broadly researched during endoplasmic reticulum (ER) stress, where it composes a critical pro-survival signaling pathway of the unfolded protein response (UPR). During UPR, the kinase, IRE1α, oligomerizes, autophosphorylates, and its endoribonuclease activity catalyzes the alternative splicing of X-box binding protein 1 (XBP1) to form an active transcription factor (XBP1s) (9). An increasing number of publications show that UPR signaling pathways are directly linked to inflammatory cytokine production (10). IRE1α has been shown to activate pro-inflammatory pathways and induces inflammatory cytokines secretion, particularly for IL-6 (11). XBP1s have been shown to bind directly to the promoters of IL-6 genes and regulate their expression (12). IRE1α has also been found to induce IL-6 expression by activating the transcription factors of NF-kB or AP-1 (13). IRE1α-induced IL-6 has been implicated in promoting carcinogenesis of HCC and proliferation of melanoma (14). A recent study revealed that inhibition of IRE1α endonuclease activity significantly reduced the growth of prostate cancer cells both *in vitro* and *in vivo* (15). All those results indicated that IRE1α plays a crucial role in PCa progression. However, the roles of IRE1α in the CRPC progression have not been fully investigated.

In the present study, we evaluated the expression of IRE1α in hormone-naïve prostate cancer tissues, CRPC tissues, and prostate cancer cell lines and investigated the effect of IRE1α expression on prostate cancer proliferation under the androgen-deficient condition *in vitro* and *in vivo*. Furthermore, we explored the potential mechanisms underlying the impact of IRE1α expression on CRPC progression. Our findings may provide a novel mechanism of CRPC progression and suggest a potential target for the prevention and treatment of CRPC.

## Materials and Methods

### Human Prostatic Tissues

A prostate cancer tissue microarray (TMA) contained 10 cores of adjacent normal tissue, 80 cores of hormone naïve prostate cancer, and 9 cores of castration-resistant prostate cancer was constructed for detecting the expression of IRE1a in prostatic tissues. Hormone-naive prostate cancer tissues and adjacent normal tissues were derived from radical prostatectomy specimens of localized prostate cancer patients at department of Urology of Tangdu hospital between 2017 and 2019. The information of these 80 prostate cancer specimens is described in **Supplementary Table S1**. CRPC tissues were collected from 9 patients who died of CRPC and underwent rapid autopsy.

### Western Blot and Immunohistochemistry

Prostate cancer tissues and cell lines were prepared for western blot and IHC, as previously described (16). Immunohistochemical staining was assessed and scored by three independent pathologists who were blinded to clinical characteristics. Pictures of three typical fields of vision were captured at high magnification (× 200), and identical acquisition settings were used for all image acquisitions. The mean density was quantified as the mean ratio of the integrated optical density of all positive staining to the total area of each photograph in five spots for each section. Median values were used as the cutoff to classify patients into high or low expression group. The kinds and working concentrations of the primary antibodies used in this study were listed in **Supplementary Table S2**.

### Quantitative Real-Time Reverse Transcription PCR (qRT-PCR)

Trizol reagent was used to extract total RNA and reverse-transcripted to cDNA using an oligo(dT) primer. PCR amplification was performed following the manufacturer’s instructions of the SYBR Green PCR Kit (Takara, 639676). The^2−ΔΔct^ method was used to quantify the relative expression level of the examined genes. The mRNA level of the specific transcripts was normalized to β-actin RNA transcripts. Key PCR primers used in this study are listed in **Supplementary Table S3**.

### Cell Lines and Cell Culture

Human prostate cancer cell lines LNCaP, VCaP, PC3, and C4-2B were purchased from the American Type Culture Collection. DU145 and LAPC4 cell lines were purchased from the Shanghai Cell Bank of the Chinese Academy of Sciences (Shanghai, China). PC-3, VCaP, and LNCaP cells were cultured in RPMI-1640 medium containing 10% FBS. Dulbecco’s Modified Eagle Medium (DMEM) was used as the culture medium for prostate cancer cells of DU-145, C4-2B, and LAPC4. All cell lines have recently been authenticated by using STR-analysis. Fetal bovine serum (FBS) and charcoal-stripped fetal bovine serum (CSS) were purchased from Omega Scientific (Tarzana, CA). Recombinant human IL-6 and neutralizing anti-human IL-6 antibody were purchased from R&D Systems Inc. (Minneapolis, MN, USA). MKC8866 and thapsigargin (TG) were purchased from Selleck Chemicals (Shanghai, China).

### RNA Silencing and Construction of Cell Lines With IRE1α or AR Knockdown or Overexpression

The empty vector pcDNA3.1 (vector), IRE1α-overexpressing vector (pcDNA3.1-IRE1α) AR-overexpressing vector (pcDNA3.1-AR), siRNA control, siRNA IRE1α, siRNA AR, and Lentivirus constructing of IRE1α knockdown or overexpression were purchased from Genechem (Obio Technology Corp, China). Prostate cancer cells plated on six-well dishes were allowed to reach 70% confluence. Prostate cancer cells were transfected with plasmids or siRNA using Lipofectamine 3000 Transfection Reagent (Invitrogen). Stable clones were obtained by selection in 400 µg/ml G418 for 2 weeks. The sequences of siRNA for IRE1α and AR are provided in **Supplementary Table S3**.

### Proliferation Assay

MTS assay was performed to evaluate cell proliferation. Prostate cancer cells were seeded in triplicate in 96-well plates at the density of 3 × 10^3^ cells per well in 100 μ L. 24 h later, the culture medium was removed and replaced with a fresh culture medium containing charcoal-stripped serum (CSS). The MTS reagent, prepared following the instruction, was added to the cells and cultured at 37°C for 2 hours. The absorbance of each well was read at wavelength 490 nm. Results were expressed as a percentage of the untreated control cells. Values were given as mean ± standard deviation (SD) of three tests.

### Colony Formation Assay

Cell growth was assessed using the colony formation assay. Prostate cancer cells with IRE1α overexpression or knockdown and the control cells were seeded at the density of 1×10^3^ cells/well in 3 mL medium supplemented with CSS in 6-well plates. The culture medium was changed every two days. After 14 days of culturing at 37°C, the colonies were fixed using 4% formaldehyde for 10 minutes and were stained with 10% Giemsa for 20min. Colonies were then washed with running water and air-dried. Finally, the images of the colonies were acquired using an Olympus digital camera.

### Human Prostate Cancer Xenografts

Intact male SCID mice were inoculated subcutaneously with prostate cancer cells with IRE1α overexpression or knockdown or control cells implanted bilaterally in 50% PBS + 50% matrigel. A vernier caliper was used to measure the tumor length (L) and width (W) weekly, and the tumor volume was estimated according to the formula (L×W^2^)/2. All mice were castrated when tumor volumes reached 200 mm^3^. 24 days after castration, the mice were sacrificed to harvest and weigh the tumors. This study obtained ethical clearance from the Animal Research Ethics Committee of the Fourth Military Medical University.

### HE Staining

HE staining of the pathological sections of the xenograft tumor tissues was performed following the instruction strictly (Beyotime, China). Representative images were acquired with an Olympus microscope (IX81; Olympus) at 200×magnification.

### Luciferase Reporter Gene Assay

Prostate cancer cells with IRE1α overexpression or knockdown were seeded 24-well plates at 3×10^4^ cells per well. After 24h, PSA-luciferase-plasmid and Renilla luciferase pRL-TK plasmid were cotransfected into prostate cancer cells using Lipofectamine 3000. 48 hrs later, cells were harvested, and luciferase activity was determined using the Dual Luciferase Reporter Assay Kit (Promega). The relative luciferase activity was normalized to the renilla luciferase activity.

### ELISA for IL-6

The secretion of IL-6 was detected using a human IL-6 ELISA kit (R&D Systems; Minneapolis, MN) following the kit instruction. Briefly, prostate cancer cells with IRE1a overexpression or knockdown were cultured in a medium containing charcoal-stripped serum (CSS). After 24 h, The cell-free culture supernatants were harvested to determine the expression levels of IL-6. The wells were coated with a capture antibody and incubated with the standard or supernatant samples. After the addition of TMB substrate, and H_2_SO_4_, the absorbance was read at 450 nm wavelength.

### Statistical Analysis

All the experiments were replicated three times. Data were plotted as the mean ± standard error of the mean (SEM). SPSS statistical software 21.0 was used for statistical analysis. p < 0.05 was considered statistically significant. Statistical comparisons were performed using paired or unpaired Student t-tests where appropriate. The Spearman rank correlation test analyzed correlations between measured variables.

## Results

### IRE1α Is Upregulated in CRPC Tissues and Cell Lines

**Figure 1:**
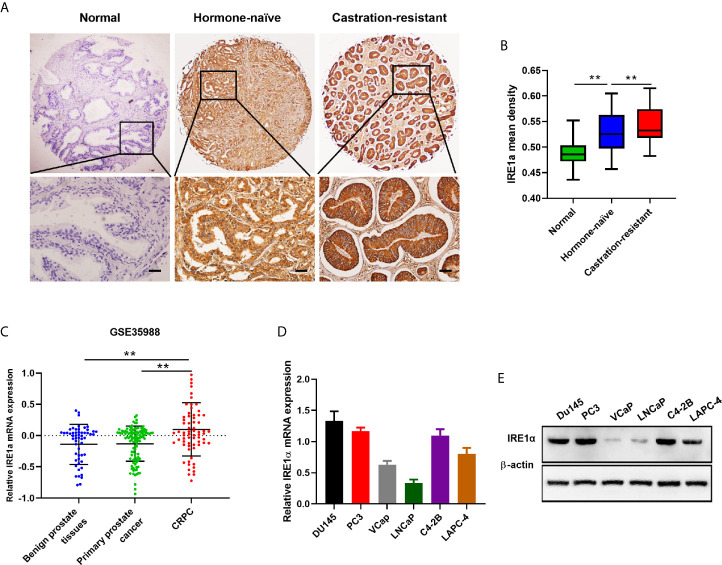
IRE1α is upregulated in CRPC tissues and cell lines. **(A, B)** Representative IHC staining images and mean IHC scores of IRE1α in adjacent non-cancerous tissues, hormone naïve prostate cancer and CRPC tissues (n=80; Scale bars, 200μm; **P < 0.01). **(C)** Expression profile of IRE1α was obtained from a GEO dataset (GSE35988). **(D, E)** qPCR and western blot analysis for the mRNA and protein expression of IRE1α in prostate cancer cell lines.

To analyze the relationship between IRE1α expression and CRPC progression, immunohistochemical (IHC) staining was performed to detect the IRE1α expression in CRPC, hormone-naïve prostate cancers and adjacent normal tissues. The IHC results showed that IRE1α expression was significantly elevated in CRPC tissues as compared to the hormone-naïve prostate cancers tissues and jacent normal tissues, the latter displayed comparably weak or negative IRE1α expression (**Figures 1A, B**). Increased IRE1α expression was also found in high Gleason score hormone-naïve prostate cancer compared to low Gleason score hormone-naïve prostate cancers (**Supplementary Figures S1A, B** and **Supplementary Table S1**). For further confirmation, we analyzed the expression of IRE1α in prostate cancer using data from the TCGA database (GSE35988). The result showed that the expression of the IRE1α gene was significantly higher in CRPC than in primary prostate cancer or benign prostatic hyperplasia (**Figure 1C**). qPCR analysis on prostate cancer cell lines revealed that IRE1α presented higher mRNA levels in several androgen-independent prostate cancer cell lines C4-2B, PC3, and DU145, as compared to androgen-dependent prostate cancer cell lines LNCaP, LAPC-4, and VCaP (**Figure 1D**). Western blot analysis demonstrated the same expression pattern of IRE1α at the protein level (**Figure 1E**). Notably, the expression of IRE1α in C4-2B cells, which is derived from a bone metastasis clone of LNCaP cells and is androgen-independent, was significantly increased than their parental androgen-dependent LNCaP cells (**Figures 1D, E**). All these results implied that the high expression level of IRE1α was tightly related to the progression of CRPC.

### Increased IRE1α Expression Promotes Prostate Cancer Cell Proliferation Under the Androgen-Deficient Condition

**Figure 2:**
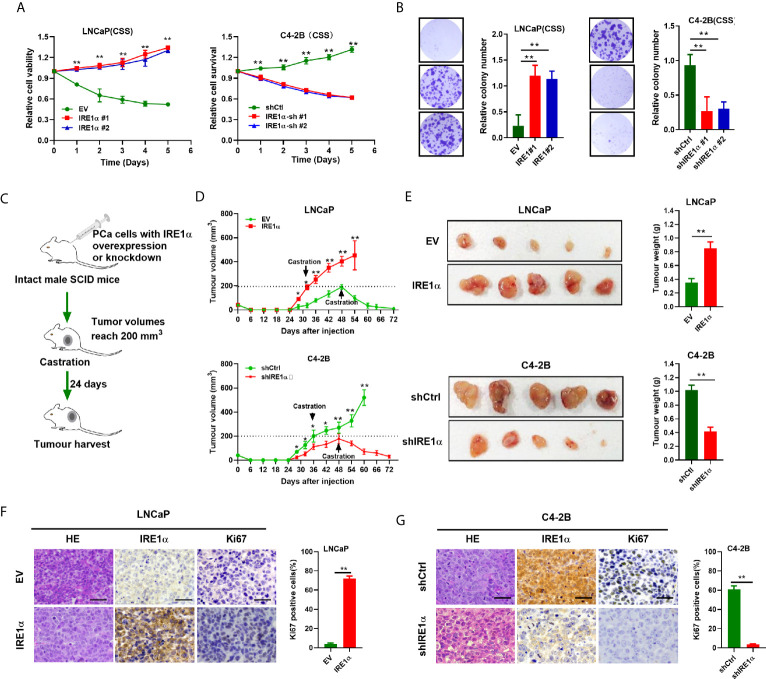
IRE1α overexpression promotes prostate cancer cells proliferation under the androgen-deficient condition. **(A, B)** MTS assay and colony formation assay of LNCaP cells with IRE1α overexpression or C4-2B cells with IRE1α knockdown, which were cultured in conditions with CSS. **(C)** The *in vivo* experiments investigated the effect of IRE1α expression on the proliferation abilities of prostate cancer cells in non-castrated and castrated male SCID mice. **(D, E)** Growth curve and tumors volumes shows the growth patterns of tumors formed by LNCaP cells with IRE1α overexpression or C4-2B cells with IRE1α knockdown, first grown in intact mice, followed by another 24 days postcastration in the same hosts. **(F, G)** Representative HE and IHC staining images of IRE1α and Ki-67 in xenograft tumor treated as indicated (Scale bar, 100μm). shCtrl, negative control short hairpin RNA; shIRE1α, short hairpin RNA against IRE1α; EV, cells transfected with empty vector; IRE1α, cells transfected with IRE1α expression vector. Data shown are the mean ± SD from three independent experiments, **P < 0.01.

To further explore the functions of IRE1α in CRPC progression, we investigated the effect of IRE1α expression on prostate cancer cell proliferation. Androgen-dependent LNCaP cells (relatively low expression of IRE1α) or androgen-independent C4-2 cells (relatively high expression of IRE1α) were selected for the establishment of cell models with forced expression or knockdown of IRE1α, respectively (**Supplementary Figures S2A, B**). MTS assays showed that when cultured in conditions with regular FBS, LNCaP cells with IRE1α overexpression proliferated more rapidly than control cells. In contrast, the growth rate of C4-2B cells with IRE1α knockdown was significantly reduced compared with control cells (**Supplementary Figure S2C**). Intriguingly, the results of MTS and colony formation assays indicated that when cultured in the androgen-deficient medium (CSS), LNCaP cells with IRE1α overexpression showed significant resistance to androgen-deprivation, as compared to control cells which failed to divide under androgen deprivation conditions. In contrast, C4-2B cells with IRE1α knockdown displayed significantly decreased proliferation rate compared with control cells, which grew aggressively even under androgen deprivation conditions (**Figures 2A, B**).

*In vivo* tumorigenicity study (**Figure 2C**) showed that LNCaP cells with IRE1α overexpression formed larger xenograft tumors faster than control cells in intact SCID mice. In contrast, xenografted tumors derived from C4-2B cells with IRE1α knockdown grown significantly slower than control xenograft tumors in intact SCID mice (**Figure 2D**). As expected, tumors formed by LNCaP cells with IRE1α overexpression did not respond to the castration of host mice and displayed a sustained proliferation; in strong contrast, tumors derived from control cells stopped growing or started to shrink in castrated hosts. Whereas the growth of C4-2B cells with IRE1α knockdown was significantly impaired compared with that of control cells, which proliferated persistently after castration (**Figures 2D, E**). Moreover, tumor proliferation was determined by Ki-67 staining *via* IHC. The results showed that the proportion of Ki-67 positive cells dramatically elevated in tumors derived from LNCaP cells with IRE1α overexpression in comparison with controls. In contrast, the tumors developed from C4-2B cells with IRE1α knockdown displayed significantly reduced Ki-67-staining cells than controls (**Figures 2F, G**). All those results suggested that elevated IRE1α expression promotes prostate cancer cell proliferation under androgen-depleted condition.

### Increased IRE1α Expression Induces Androgen Receptors Activation

**Figure 3:**
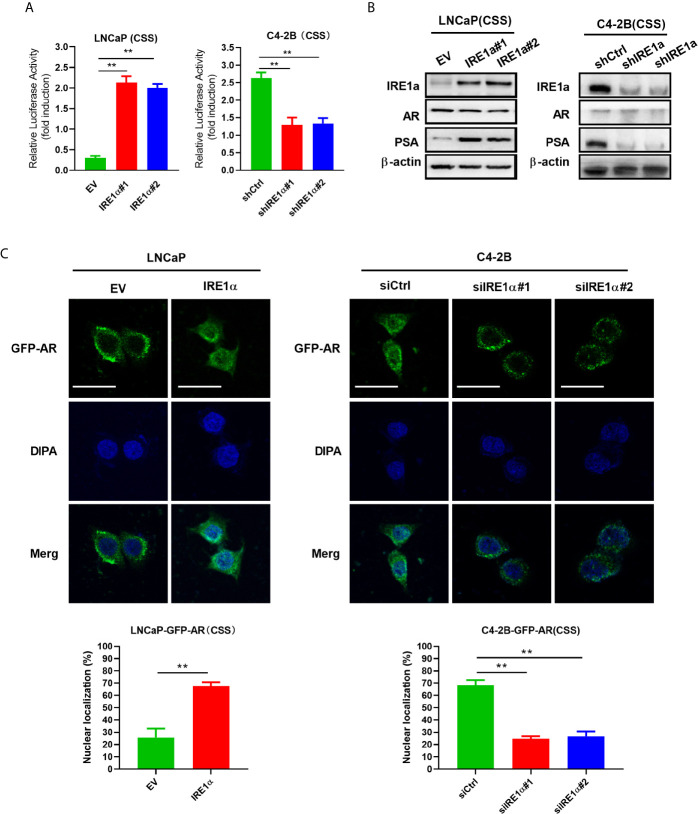
Overexpression of IRE1α induces androgen receptor activation. **(A)** IRE1α expression affects AR transcriptional activity. LNCaP cells with IRE1α overexpression or C4-2B cells with IRE1α knockdown were transiently transfected with PSA-Luc and then cultured in CSS medium. Luciferase activity was assayed 48 h after transfection. **(B)** IRE1α expression affects AR activation without affecting the AR level. Representative western blot analyses of the expression of IRE1α, AR, and the AR target gene PSA in LNCaP cells with IRE1α overexpression or C4-2B cells with IRE1α knockdown. **(C)** IRE1α expression affects the subcellular localization of GFP-AR. LNCaP cells and C4-2B cells stably expressing GFP-AR were transiently transfected with empty vector (EV) or IRE1α expression vector (IRE1α), control siRNA (siCtrl) or IRE1α siRNA, and then cultured in the medium with of CSS for 48h, confocal microscopy was used to visualize the subcellular localization of GFP-AR. Data shown are the mean ± SD from three independent experiments, **P < 0.01.

Statistical analysis showed that IRE1α expression was significantly associated with the serum levels of PSA, a well-known androgen receptor target gene (**Supplementary Table S1**). Since the proliferation and survival of prostate cancer heavily depend on AR even in the absence of androgen, we asked whether IRE1α expression has effects on AR activation. To do so, prostate cancer cells with IRE1α overexpression or knockdown were transiently transfected with a PSA-driven luciferase reporter (PSA-LUC) and cultured in the androgen-deficient medium(CSS). As shown in **Figure 3A**, the overexpression of IREα led to a substantial ligand-independent increase in PSA-LUC activity in LNCaP cells. On the contrary, IRE1α knockdown in C4-2B cells significantly decreased the luciferase activity of PSA-LUC under androgen deprivation conditions (CSS) (**Figure 3A**). Western blot analyses were performed to analyze the effects of IRE1α expression on the protein expression of AR and PSA. The results showed that IRE1α overexpression or knockdown had no detectable impact on AR levels, while the levels of PSA greatly increased with IRE1α overexpression and significantly decreased with IRE1α knockdown (**Figure 3B**). Furthermore, we used LNCaP and C4-2 cells, stably expressing GFP-AR, as a model system to test the impact of IRE1α expression on AR subcellular localization. The results showed that transient overexpression of IRE1α led to significant accumulation of GFP-AR in the nucleus of LNCaP cells cultured under androgen deprivation conditions (CSS). In contrast, the knockdown of IRE1α by siRNA in C4-2B cells significantly inhibited the nuclear translocation of GFP-AR (**Figure 3C**).

### Increased IRE1α Expression Promotes Interleukin-6 Secretion *via* the IRE1a/XBP-1s Pathway in Prostate Cancer Cells

**Figure 4:**
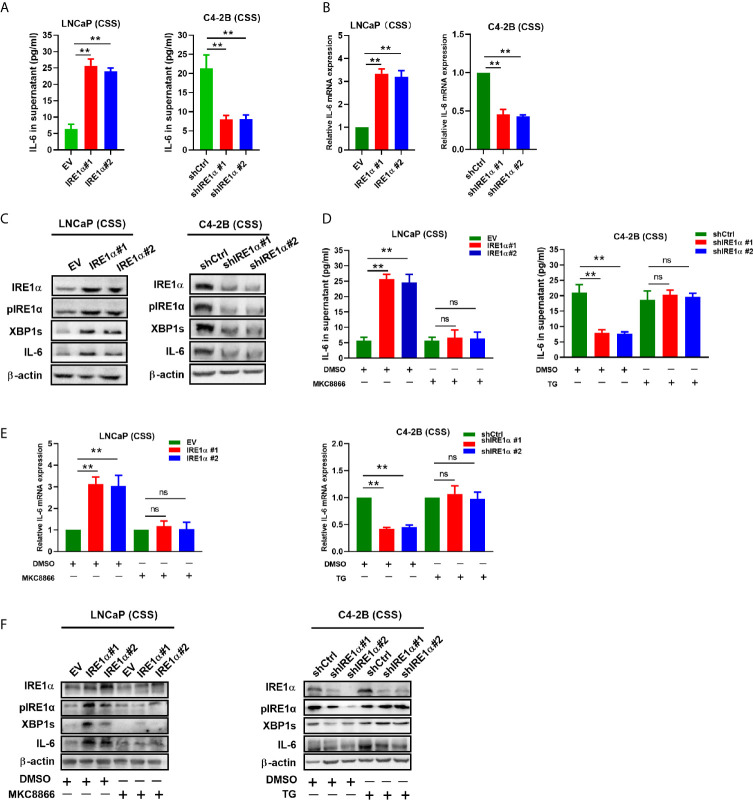
IRE1α stimulates IL-6 production *via* the IRE1α/XBP-1s pathway in prostate cancer cells. **(A)** LNCaP cells with IRE1α overexpression or C4-2B cells with IRE1α knockdown were cultured in an androgen deprivation medium with CSS for 48 h, and mRNA levels of IL-6 were determined by qPCR. **(B, C)** ELISA and western blot analysis for the protein expression of IL-6 in LNCaP cells with IRE1α overexpression or C4-2B cells with IRE1α knockdown. **(D–F)** MKC8866 and thapsigargin (TG), the inhibitor and the agonist of the IRE1α/XBP-1s pathway, were used to detect the effect of the IRE1α/XBP-1s pathway on IL-6 expression. Data shown are the mean ± SD from three independent experiments, ^**^P < 0.01. ns, no significance.

IRE1α has been reported to stimulate IL-6 secretion *via* the IRE1α/XBP-1s pathway (17). Here, we investigated whether IRE1α can induce IL-6 secretion *via* the IRE1α/XBP-1s pathway in prostate cancer cells. Firstly, we assessed the effect of IRE1α expression on IL-6 expression. To achieve this, prostate cancer cells with IRE1α overexpression or knockdown were cultured with the androgen-deficient medium (CSS) for 48h, and the cell culture supernatant was collected for IL-6 ELISA. The results demonstrated that the level of IL-6 was significantly elevated in the culture supernatant of LNCaP cells with IRE1α overexpression than that of the control cells. In contrast, C4-2B cells with IRE1α knockdown showed significantly decreased secreted IL-6 levels as compared with the control group (**Figure 4A**). The promoting effect of IRE1α on IL-6 secretion was further confirmed by qPCR at mRNA levels (**Figure 4B**). Western blot results showed that the protein levels of phosphorylated IRE1α, XBP-1s, and IL-6 increased with the overexpression of IRE1α and decreased with IRE1α knockdown (**Figure 4C**). Moreover, MKC8866, an inhibitor of the IRE1α/XBP-1s pathway, significantly reduced the relative expression levels of IL-6 at both mRNA and protein levels, which was increased dramatically by IRE1α overexpression in LNCaP cells (**Figures 4D–F**, left). Conversely, thapsigargin (TG), an agonist of the IRE1α/XBP-1s pathway, restored IL-6 expression significantly decreased by IRE1α knockdown in C4-2B cells. Altogether, these results indicated that IRE1α affected IL-6 expression *via* the IRE1α/XBP-1s pathway (**Figures 4D–F**, right).

### IL-6 Mediated IRE1α Induced AR Activation

**Figure 5:**
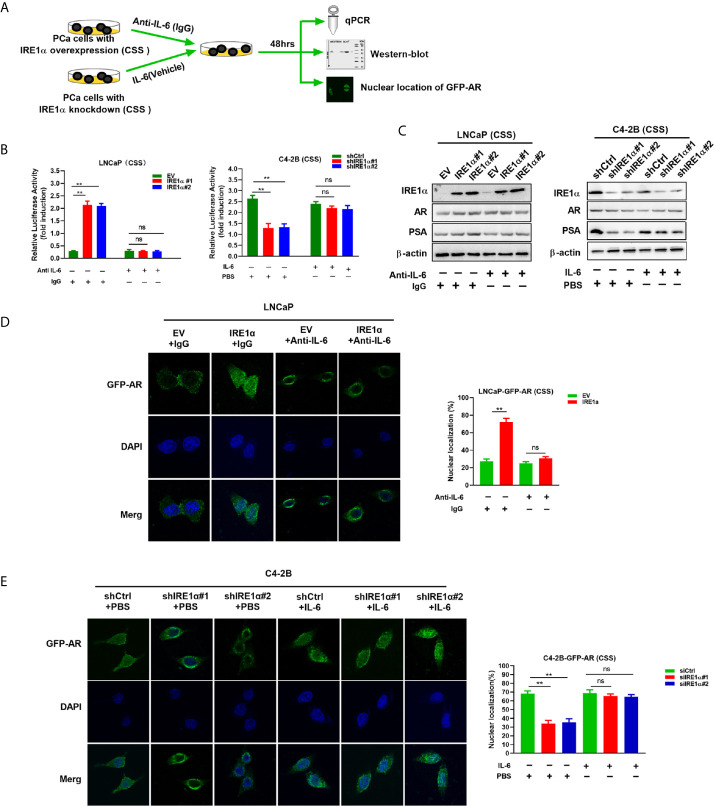
IRE1α activates AR by inducing IL-6 secretion. **(A)** LNCaP cells with IRE1α overexpression and C4-2B cells with IRE1α knockdown were treated with anti-IL-6 antibodies or IL-6 respectively, and AR transcriptional activity was measured by luciferase reporter assay, western-blot and nuclear localization of GFP-AR after 48 hours of treatment. **(B)** LNCaP cells with IRE1α overexpression and C4-2B cells with IRE1α knockdown were transiently transfected with PSA-Luc and then treated with anti-IL-6 antibodies or recombinant IL-6, respectively. Luciferase activity was assayed and normalized. **(C)** Western blot assays were performed to determine the expression levels of IRE1α, AR, and AR target gene PSA in LNCaP cells with IRE1α overexpression or C4-2B cells with IRE1α knockdown, which have been treated with anti-IL-6 or recombinant IL-6, respectively. **(D, E)** Fluorescent microscopy was used to detect whether IL-6 affected IRE1α-induced nuclear localization of GFP-AR. Data shown are the mean ± SD from three independent experiments, **P < 0.01; ns, no significance.

It is well established that IL-6 can induce the activation of AR in a ligand-independent manner. Therefore, it is reasonable to hypothesize that IRE1α may activate AR *via* modulating the expression of IL-6. To test it, anti-IL-6 antibody or recombinant IL-6 were separately added to the androgen deprivation medium of LNCaP cells with IRE1α overexpression or C4-2B cells with IRE1α knockdown (**Figure 5A**). As shown in **Figure 5B** left, the anti-IL-6 antibody significantly attenuated the PSA-LUC activity, which was increased by IRE1α stable overexpression in LNCaP cells. In contrast, recombinant IL-6 significantly enhanced the luciferase activity of PSA-LUC, which was decreased by knockdown of IRE1α in C4-2B cells (**Figure 5B**, right). Besides, anti-IL-6 antibody significantly abrogated IRE1α overexpression-induced PSA expression and GFP-AR nuclear translocation in LNCaP cells, while the addition of recombinant IL-6 significantly restored the reduction of PSA expression and GFP-AR nuclear translocation induced by IRE1α knockdown in C4-2B cells (**Figures 5C–E**). Neither anti-IL-6 antibody nor recombinant IL-6 affected the expression levels of AR (**Figure 5C**). Taken altogether, all these indicated that IL-6 is a crucial factor implicated in the effects of IRE1α on the activation of AR.

### The Activation of AR by IL-6 Induces IRE1α Expression

**Figure 6:**
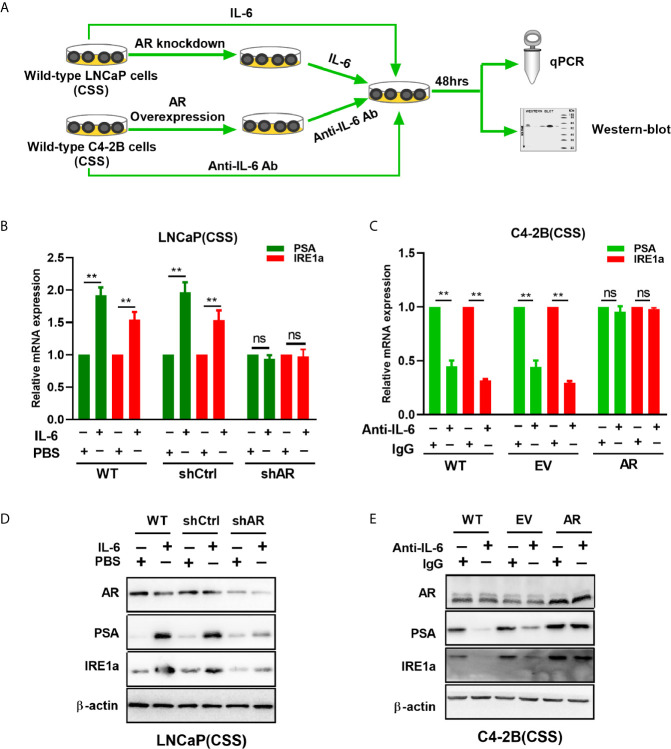
The effect of IL-6 induced AR activation on IRE1α expression. **(A)** Wild-type LNCaP cells or LNCaP cells with AR knockdown were treated with IL-6, and wild-type C2-4 cells or C2-4 cells with AR overexpression were treated with anti-IL-6 antibodies. The expression of AR target gene PSA and IRE1α were analyzed by qPCR and western blot analysis. **(B, D)** qPCR and western blot analysis were used to analyzed for the expression of AR target gene PSA and IRE1α in IL-6 treated wild-type LNCaP cells or LNCaP cells with AR knockdown. **(C, E)** qPCR and western blot analysis were used to analyzed for the expression of PSA and IRE1α in anti-IL-6 antibodies treated wild-type C4-2B cells or C2-4 cells with AR overexpression. Data shown are the mean ± SD from three independent experiments, **P < 0.01; ns, no significance.

A recent study showed that androgen-mediated AR activation could induce IRE1α expression in prostate cancer cells (18). Here, we investigated whether IL-6-mediated AR activation can regulate IRE1α expression in prostate cancer cells. To achieve this, wild-type LNCaP and C4-2B cells were cultured with the androgen-deficient medium (CSS) and treated with recombinant IL-6 or anti-IL-6 antibody, respectively (**Figure 6A**). Our results showed that IL-6 treatment significantly increased the expression of AR target gene PSA and IRE1α at both mRNA and protein levels in LNCaP cells (**Figures 6B, D**). In contrast, treatment with anti-IL-6 antibody significantly reduced the expression levels of PSA and IRE1α in C4-2B cells (**Figures 6C, E**). Those results indicated that IL-6 could induce AR activation and IRE1α expression. To further elucidate the relationship between AR activation by IL-6 and the expression of IRE1α, we knocked down the AR expression in LNCaP cells and upregulated AR in C4-2B cells (**Figure 6A**). As shown in **Figures 6B, D**, knockdown of AR significantly attenuated the mRNA and protein levels of IRE1a, which were increased by IL-6 stimulation in LNCaP cells. In contrast, overexpression of AR significantly reverted the mRNA and protein expression of IRE1α, which were decreased by anti-IL-6 antibody in C4-2B cells (**Figures 6C, E**). Collectively, these data demonstrate that AR activation by IL-6 induces IRE1α expression.

### IRE1α Promotes the Castration-Resistant Growth of Prostate Cancer Cells in an IL-6/AR-Mediated Manner

**Figure 7:**
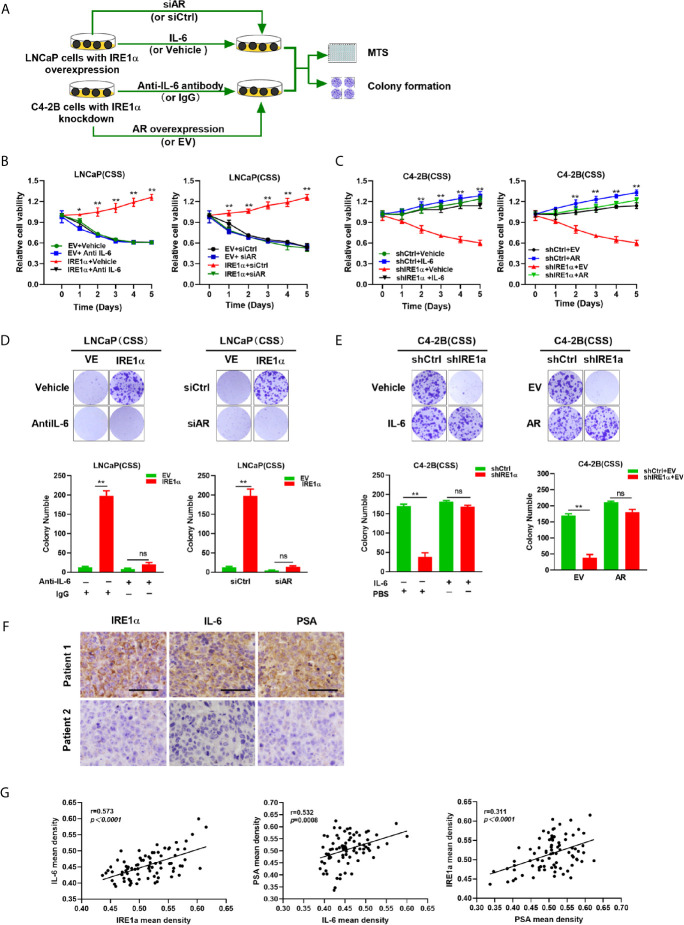
IRE1α promotes castration-resistant growth of prostate cancer cells in an IL-6/AR-mediated manner. **(A)** LNCaP cells with IRE1α overexpression were treated with anti-IL-6 antibodies or transiently transfected with AR siRNA, and C4-2B cells with IRE1α knockdown were treated with anti-IL-6 antibodies or transiently transfected with AR expression vector. MTS assay and colony formation assay were used to detect the effect of IL-6 or AR on IRE1α-induced proliferation of prostate cancer cells. **(B, D)** MTS assay and colony formation assay of LNCaP cells with IRE1α overexpression, which were treated with anti-IL-6 or transiently transfected with control siRNA or AR siRNA. **(C, E)** MTS assay and colony formation assay of C4-2B cells with IRE1α knockdown, which were treated with IL-6 or transiently transfected with AR expression vector (AR) and empty vector (EV). **(F)** Representative prostate cancer samples showing the expression of IRE1α, PSA and IL-6; patient 1, IRE1α high; patient 2, IRE1α low. Scale bar, 200μm. **(G)** Scatter plot analysis revealed that protein levels of IRE1α, PSA and IL-6 in prostate cancer tissues. Data shown are the mean ± SD from three independent experiments, **P < 0.01; ns, no significance.

Our data indicated that IRE1α, IL-6 and AR formed a positive feedback loop, and through which facilitated prostate cancer cell proliferation under androgen deprivation conditions. To confirm this hypothesis, LNCaP cells with IRE1α overexpression were cultured in the androgen-deficient medium and treated with anti-IL-6 antibody or transiently transfected with siCtrl or siAR. The results of MTS and colony formation assays showed that treatment with anti-IL-6 antibody or knockdown of AR significantly attenuated the proliferative and clone formation capability, which was increased by IRE1α overexpression in LNCaP cells (**Figures 7A, B**, **D**). In contrast, the addition of IL-6 or transiently transfected with an overexpression plasmid for AR significantly restored prostate cancer cell proliferation and colony formation, which was reduced by knockdown of IRE1α in C4-2B cells (**Figures 7A, C, E**). To further support these results, we measured the expression of IL-6 and PSA in prostate cancer tissues by IHC and analyzed the relationship between the expression levels of IRE1α, IL-6 and PSA. Representative cases are shown in **Figure 7F**. Prostate cancer patients with higher IRE1α expression also had a higher IL-6 or PSA expression, and vice versa. Spearman rank correlation analysis revealed significant positive correlation between mean IHC scores of IRE1α and IL-6 (r = 0.573, p<0.0001), PSA and IL-6 (r = 0.532, p=0.0008) or IRE1α and PSA (r = 0.311, p<0.0001) (**Figure 7G**). All those results indicated that IRE1α promotes the progression of prostate cancer in an IRE1α-IL-6-AR-positive feedback loop manner.

## Discussion

**Figure 8:**
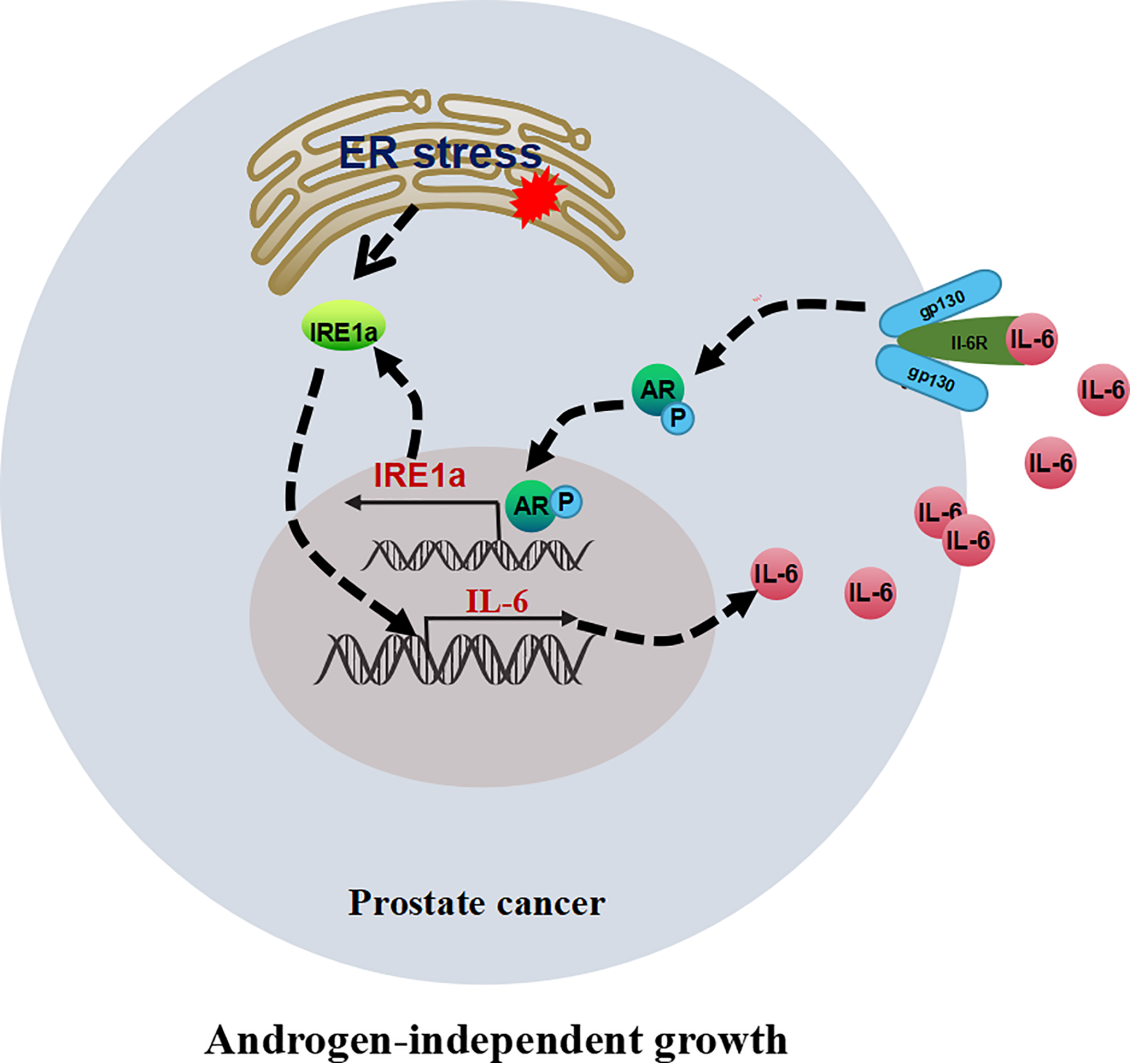
The proposed model illustrates the positive feedback loop between IRE1α, IL-6, and AR in controlling the proliferation of prostate cancer cells.

In this study, we demonstrated that the expression of IRE1α was significantly increased in CRPC tissues and androgen-independent prostate cancer cell lines. Increased IRE1α expression facilitated prostate cancer cell proliferation under the androgen-deficient condition *in vivo* and *in vitro*. Mechanistically, IRE1α overexpression activated AR *via* inducing IL-6 expression. The activation of AR by IL-6 in turn stimulated IRE1α expression. IRE1α formed a positive feedback loop with IL-6 and AR to promote CRPC progression (**Figure 8**).

Ectopic expression of IRE1α has been reported in multiple types of cancer. For instance, Zheng et al. reported that IRE1a protein expression is increased in colorectal cancer (CRC) tissues, and excessive IRE1α expression is associated with reduced overall survival of patients with CRC (19). Liu et al. reported that increased IRE1α expression is observed in HCC tissues and is significantly associated with the poor prognosis of HCC patients (20). Lu et al. found that overexpression of IRE1α promotes the development of resistance in NSCLC cells (21). Consistent with previous research, our study showed that the expression of IRE1α significantly increased in the tissues of prostate cancer, CRPC, and androgen-independent prostate cancer cell lines, suggesting that increased IRE1α expression played a crucial role in CRPC progression. To the best of our knowledge, this study is the first to report the correlation between IRE1α expression and CPRC progression.

IRE1α has been implicated in the regulation of cellular proliferation. The increased expression level of IRE1α has been shown to enhance the proliferation of pancreatic islet and hepatocyte cells (22). Moreover, high expression of IRE1α has been reported to enhance the proliferation of melanoma cells (14). The knockdown of IRE1α significantly inhibited colon cancer cell proliferation *in vivo* and *in vitro* (19). The recent studies demonstrated that IRE1α plays an essential role in promoting prostate cancer cell survival, and specific inhibition of the RNase activity of IRE1α can significantly inhibit prostate cancer growth (15). IRE1α has also been shown to enhance prostate cancer cell proliferation through inducing cyclin A1 expression (23). All these data suggested that IRE1α plays a vital role in regulating prostate cancer growth. However, the relationship between IRE1α expression and the CRPC growth remains unclear. The data we presented here showed that IRE1α overexpression enhanced the proliferation ability of prostate cancer cells under the androgen deprivation conditions *in vivo* and *in vitro*. Our results strongly suggest that IRE1α may be a potential therapeutic target for CRPC.

Studies have suggested that the reactivation of the androgen receptor (AR) was the primary driver of CRPC progression (3). Several mechanisms have been implicated in the reactivation of AR in CRPC, including AR gene amplification, AR gene mutations, constitutively active AR-splice variants, enhanced co-regulators to the AR, and cytokine-induced AR activation, etc. Among them, cytokine-induced AR activation has received increasing attention in recent years. IL-6 is a well reported cytokine volved in regulating AR activation. IL-6 has been shown to activate AR in a non-ligand-dependent manner (24). Studies have demonstrated that IL-6 activates AR-signaling through mechanisms of enhancing AR-ARE DNA binding activity, promoting AR nuclear translocation, or activating a Stat3 pathway (25). IL-6-induced AR activation has been displayed to play an important role in the transition of the androgen-dependent to the androgen-independent prostate cancer (24). IL-6 has been shown to promote androgen-dependent prostate cancer cell proliferation under low-androgen conditions *in vitro* and in castrated mice, accompanied by PSA gene expression (26). IRE1α has been shown to induce IL-6 production by activating the transcription factors nuclear factor κB (NF-κB) and activator protein 1 (AP-1). Also, IRE1α-induced XBP1s can directly bind to the promoter of IL-6 and induce IL-6 expression (27). Ectopic expression of IRE1α or XBP1s robustly enhances the expression and secretion of IL-6 in hepatocellular carcinoma and melanoma cells (28). Here, we demonstrated that IRE1α overexpression significantly induced the secretion of IL-6 *via* the IRE1α/XBP-1s pathway, and IRE1α-induced IL-6 can activate AR in a ligand-independent manner. Our findings may provide a novel mechanism of AR reactivation in prostate cancer.

Previous studies have demonstrated that androgen-induced AR activation can induce UPR-related gene expression (18). Using gene expression profiling strategies, Takehiko et al. found that treatment of prostate cancer cells with androgen leads to ER stress-associated gene expression (29). Also, AR has been demonstrated to directly bind to the regulatory regions of both IRE1α and XBP-1 and regulate their expression in prostate cancer cells. Furthermore, analysis of prostate cancer gene expression datasets showed that there was a significant positive correlation between the expression levels of the AR target gene and IRE1α-related gene expression (30). Here, we revealed that treatment with IL-6 significantly increased the expression of both PSA and IRE1α in wild-type LNCaP cells, and this promoting effect was abolished by AR knockdown. In contrast, the anti-IL-6 antibody significantly decreased the expression of both PSA and IRE1α in wild-type C4-2B, and this suppressive function was restored by AR overexpression. All these indicated that IL-6 promoted IRE1α expression in an AR activity-dependent manner. Here, AR expression regulation was used instead of AR activity regulation because the changes of AR activity were consistent with the trend of the expression level of AR to some extent (31, 32).

Our data showed that IRE1α overexpression activated AR *via* inducing IL-6 secretion, and the activation of AR by IL-6 in turn promoted IRE1α expression, thus creating the IRE1α/IL-6/AR positive feedback loop. IRE1α overexpression facilitated prostate cancer cell proliferation under the androgen-deficient condition by mediating this positive feedback loop. The role of the IRE1α/IL-6/AR positive feedback loop in controlling castration-resistant growth of prostate cancer cells suggests that this signaling network could be a prognostic indicator and therapeutic target of CRPC. In our prostate cancer patient cohort, we showed that the protein expression of IRE1α, IL-6, and AR correlated in prostate cancer tissues, which may confirm the existence of IRE1α/IL-6/AR positive feedback loop in prostate cancer tissues. Indeed, the effect of IRE1α/IL-6/AR positive feedback loop on CRPC progression required further validation *in vivo*.

Collectively, our work elucidated the potential interactions between IRE1α, IL-6, and AR activation, which revealed a new molecular mechanism of CRPC progression. Our results also suggest that the IRE1α may be an important predictive biomarker for CRPC and targeting the IRE1α/IL-6/AR loop might be an effective therapeutic strategy against CRPC.

## Data Availability Statement

The datasets presented in this study can be found in online repositories. The names of the repository/repositories and accession number(s) can be found in the article/**Supplementary Material**.

## Ethics Statement

The studies involving human participants were reviewed and approved by the ethics committee of the Fourth Military Medical University. The patients/participants provided their written informed consent to participate in this study. The animal study was reviewed and approved by Animal Research Ethics Committee of the Fourth Military Medical University.

## Author Contributions

All authors listed have made a substantial, direct, and intellectual contribution to the work, and approved it for publication.

## Funding

This work was supported by the National Natural Science Foundation of China (81672340 and 81872077) and the Natural Science Foundation of Shanxi Province (2017JM8020 and 2016JM8003).

## Conflict of Interest

The authors declare that the research was conducted in the absence of any commercial or financial relationships that could be construed as a potential conflict of interest.
