# Supplementary material for: IRE1α Expedites the Progression of Castration-Resistant Prostate Cancers via the Positive Feedback Loop of IRE1α/IL-6/AR
Source: Front Oncol. 2021 Jul 6;11:671141. doi: 10.3389/fonc.2021.671141 (PMC8290131; doi:10.3389/fonc.2021.671141)
Supplement: Supplementary file 1 [file DataSheet_1.doc]

**Supplementary information**

[**IRE1α expedites the progression of castration-resistant prostate cancers via the positive feedback loop of IRE1α/IL-6/AR**](https://pubmed.ncbi.nlm.nih.gov/25382750/)

**Supplementary Figures**

**
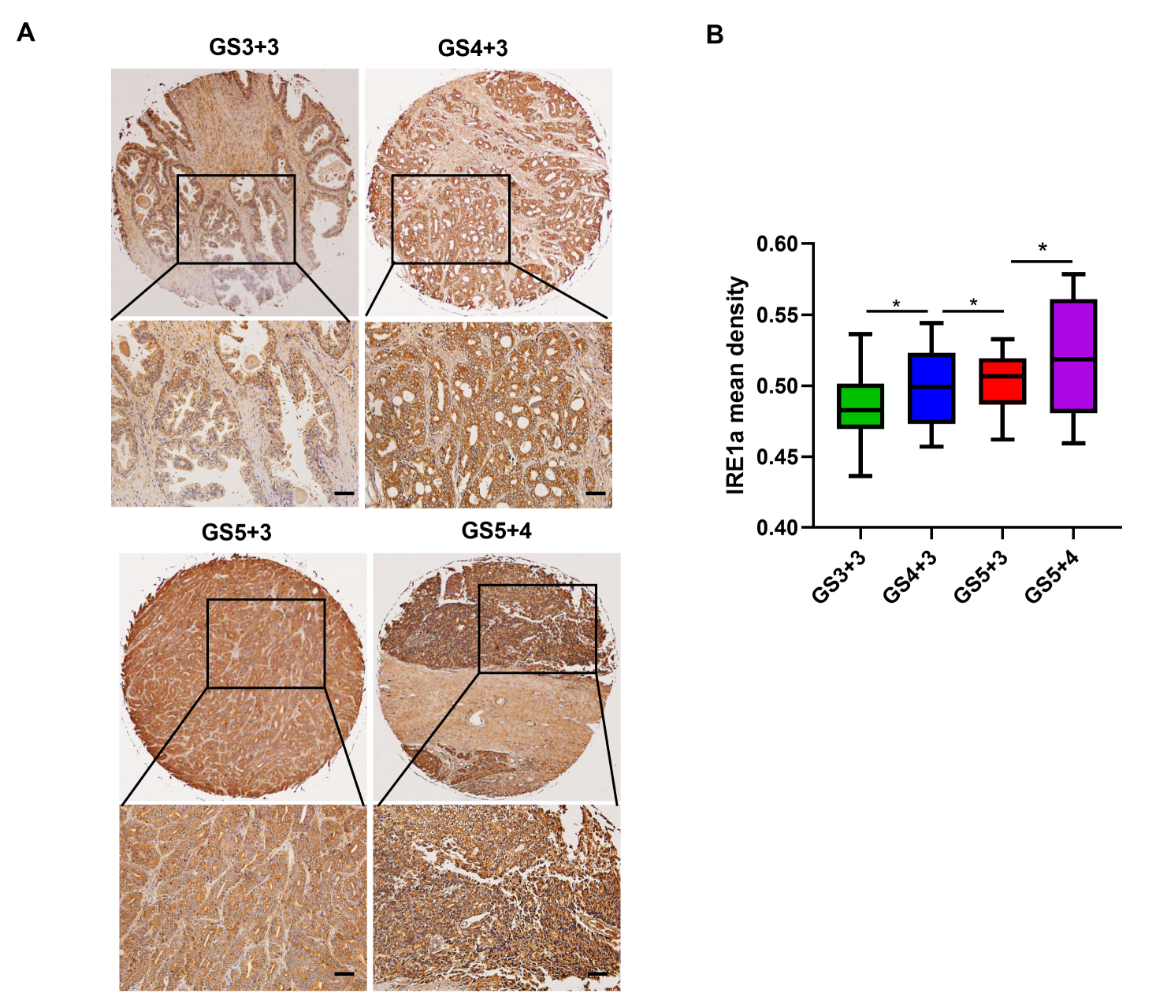
**

**Figure S1.** **Expression levels of IRE1a in prostate cancer tissues with different Gleason scores.**

A-B. Representative IHC staining images (left) and IHC scores (right) of IRE1a in prostate cancer tissues with different Gleason scores (Scale bar, 200μm).

**
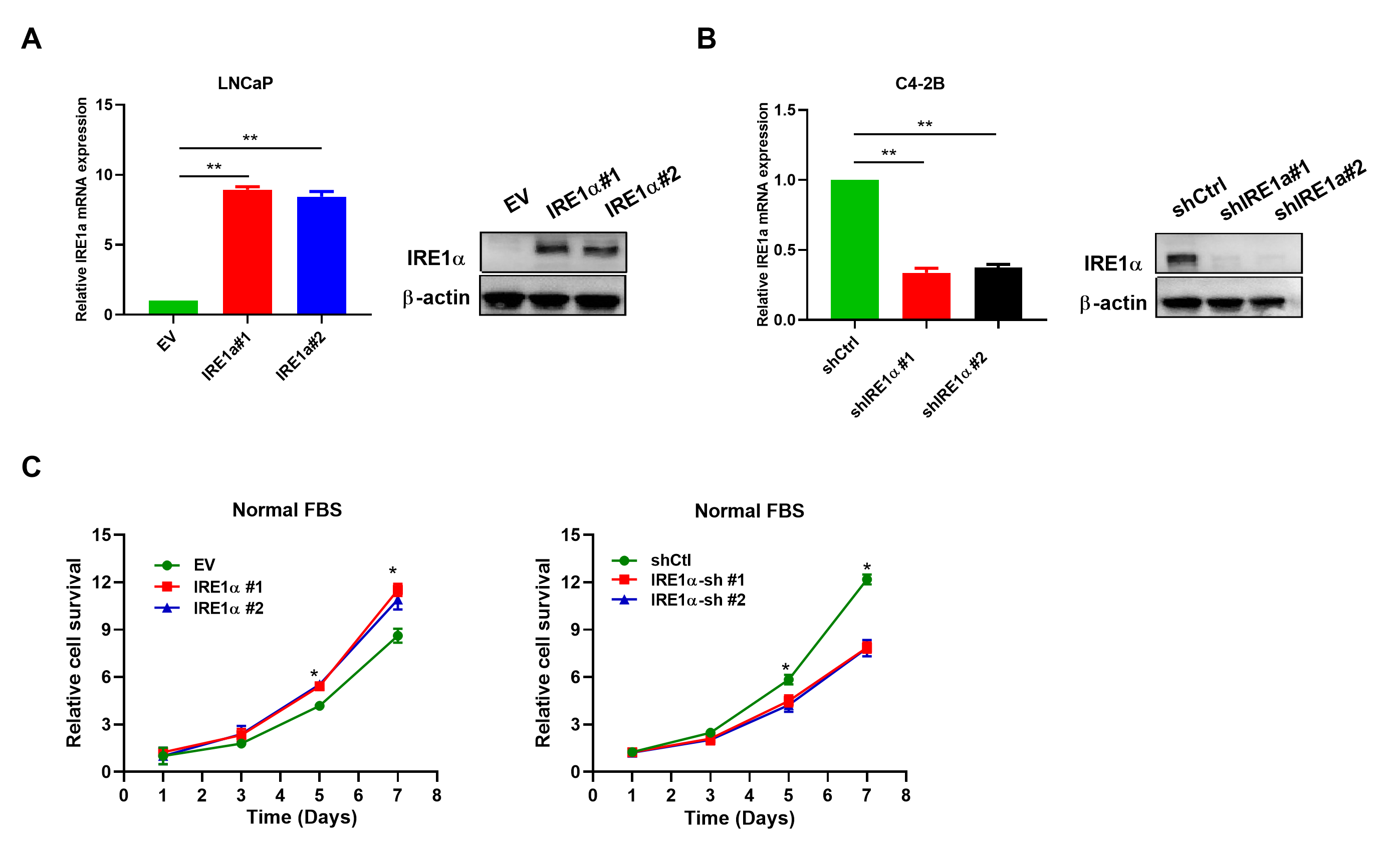
**

**Figure S2.** **Validation of IRE1a overexpression and knockdown in prostate cancer cells and the effect of IRE1a expression on the growth of prostate cancer cells cultured in normal medium.**

**A, B.** The efficacy of IRE1a overexpression and knockdown was evaluated by qPCR and western blot. **C,** MTS assay of prostate cancer cells with IRE1a overexpression or knockdown cultured in medium with normal FBS

**
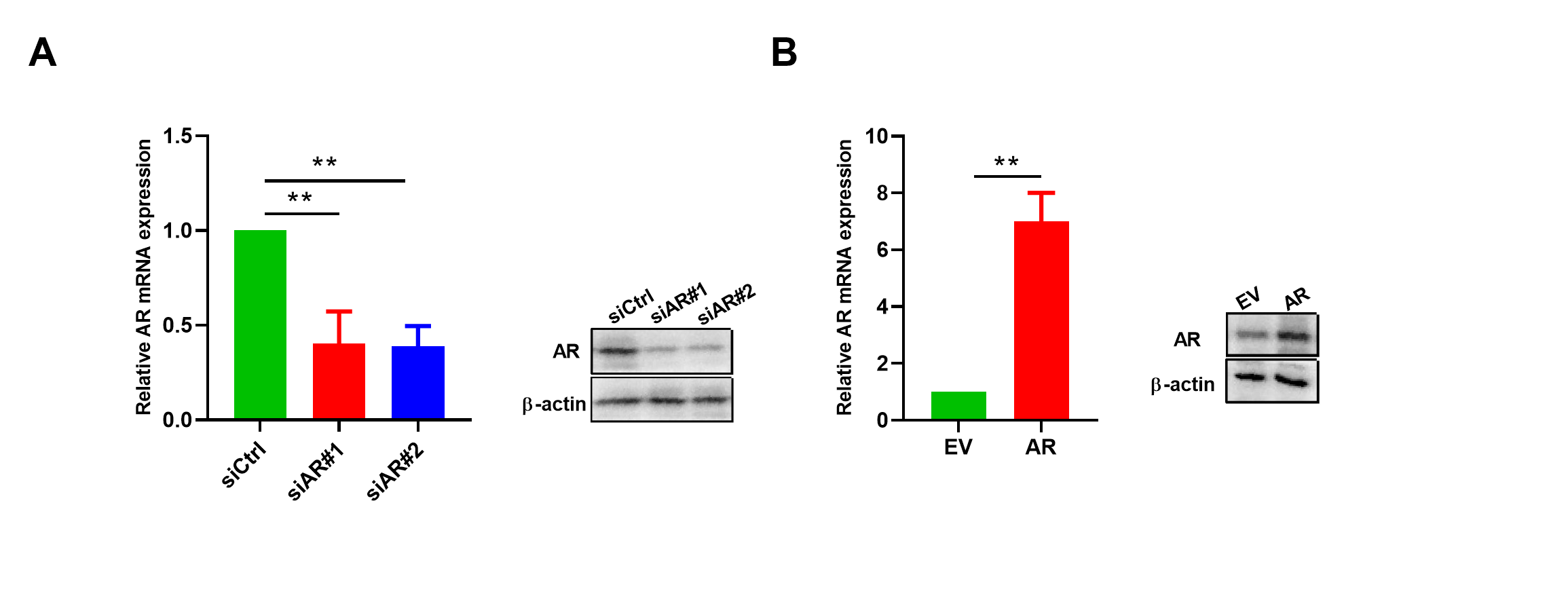
**

**Figure S3.Overexpression and knockdown efficiency of AR.**

**A, B.** The efficacy of AR overexpression and knockdown was evaluated by qPCR and western blot.

**Supplementary Table 1. | Correlation between the expression of IRE1α and the clinicopathological parameters of patients with prostate cancer.**

| **Characteristics** | **IRE1α low expression** | | **IRE1α high expression** | | *P* |
| --- | --- | --- | --- | --- | --- |
|  | N 54 | % | N26 | % |  |
| **Tumour stage** |  |  |  |  | 0.127 |
| pT2 | 28 | 51.9 | 10 | 38.5 |  |
| pT3a | 22 | 40.7 | 10 | 38.5 |  |
| pT3b-pT4 | 4 | 7.4 | 6 | 23 |  |
| **Gleason grade** |  |  |  |  | 0.034* |
| ≤3+3 | 16 | 29.6 | 3 | 11.5 |  |
| 3+4 | 28 | 51.9 | 9 | 34.6 |  |
| 3+4 Tert.5 | 2 | 3.7 | 2 | 7.7 |  |
| 4+3 | 5 | 9.2 | 7 | 26.9 |  |
| 4+3Tert.5 | 2 | 3.7 | 3 | 11.5 |  |
| ≥4+4 | 1 | 1.9 | 2 | 7.7 |  |
| **Lymph node metastasis** |  |  |  |  | 0.147 |
| N0 | 38 | 70.3 | 14 | 53.8 |  |
| N+ | 16 | 29.6 | 12 | 46.2 |  |
| **Preoperative PSA levels (ng/ml)** |  |  |  |  | 0.029* |
| ＜4 | 19 | 35.2 | 5 | 19.2 |  |
| 4-10 | 26 | 48.1 | 9 | 34.6 |  |
| 10-20 | 7 | 13.0 | 7 | 26.9 |  |
| ＞20 | 2 | 3.7 | 5 | 19.2 |  |
| **Surgical margin** |  |  |  |  | 0.370 |
| Negative | 34 | 63 | 19 | 73 |  |
| Positve | 20 | 37 | 7 | 27 |  |

***Statistically significant.**

**Supplementary Table 2. Primary antibodies used for western blotting and immunohistochemistry analysis.**

| **Antibody** | **Company (Cat. No.)** | **Working dilutions diDIConcentrationdilutions** |
| --- | --- | --- |
| IRE1a | NOVUS (NB100-2324)  IRE1 alpha Antibody) | WB: 1/1000 IHC:1:500 |
| p-IRE1α | Abcam (ab243665) | WB: 1/1000 |
| XBP-1s | Cell signaling (#40435) | WB: 1/1000 |
| β-actin | Abcam (ab8226) | WB: 1/1000 |
| IL-6 | Proteintech (66146-1-Ig) | WB : 1:1000-1:6000  IHC : 1:50-1:500 |
| PSA | NOVUS (5A11E9) | WB: 1:500-1:2000  IHC: 1:10-1:500 |
| AR | Abcamb (ab273500) | WB: 1:1000 |
| Ki67 | Abcamb (ab15580) | IHC: 1:500 |

**Supplementary Table 3. Sequences of primers and siRNAs.**

| **Primer name** | **Sequences** |
| --- | --- |
| **1. Primers for real-time PCR:** |  |
| IRE1a forward primer: | CCCAAATGTGATCCGCTACT |
| reverse primer: | TTGAGAGAATGCAGGTGTGC |
| β-actin forward primer: | ACCAACTGGGACGACATGGAG |
| reverse primer: | GTGAGGATCTTCATGAGGTAGTC |
| IL-6 forward primer: | GCACTGGCAGAAAACAACCT |
| reverse primer: | TCAAACTCCAAAAGACCAGTGA |
| PSA forward | GAGCACCCCTATCAACCCCCTATT |
| reverse | AGCAACCCTGGACCTCACACCTAA |
| **2.Primers for gene cloning** | |
| IRE1a forward primer: | CCCAAATGTGATCCGCTACT |
| reverse primer: | TTGAGAGAATGCAGGTGTGC |
| AR forward primer: | AAGGCTATGAATGTCAGCCCA |
| reverse primer: | CATTGAGGCTAGAGAGCAAGGC |
| **3. siRNAs** |  |
| IRE1a siRNA 1 sense: | CGUCGGAUGUCAUGGAUCA |
| antisense: | UGAUCCAUGACAUCCGACG |
| IRE1a siRNA 2 sense: | UCAAAGAGCUCAGUUCCAU |
| antisense: | AUGGAACUGAGCUCUUUGA |
| AR siRNA sense: | 5'-CAAGAUCCUUUCUGGGAAATT-3′ |
| antisense: | 5'-UUCUCCGAACGUGUCACGU-3' |

**Supplementary Table 4.** **Public datasets used for bioinformatics analysis.**

| **Accession No.** | **Platform** | **Probes/**  **Genes** | **HCC Sample**  **No.** | **Patient Ethnicity** | **Source URL** |
| --- | --- | --- | --- | --- | --- |
| GSE35988 | Agilent-014850 Whole Human Genome Microarray 4x44K G4112F  Agilent-012391 Whole Human Genome Oligo Microarray G4112A  Agilent-014698 Human Genome CGH Microarray 105A (G4412A) | GPL6480  GPL6848  [GPL9075](https://www.ncbi.nlm.nih.gov/geo/query/acc.cgi?acc=GPL9075) | 244 | USA | https://www.ncbi.nlm.nih.gov/geo/query/acc.cgi?acc=GSE35988 |
